# The *VHSE*-Based Prediction of Proteasomal Cleavage Sites

**DOI:** 10.1371/journal.pone.0074506

**Published:** 2013-09-09

**Authors:** Jiangan Xie, Zhiling Xu, Shangbo Zhou, Xianchao Pan, Shaoxi Cai, Li Yang, Hu Mei

**Affiliations:** 1 Key Laboratory of Dependence Service Computing in Cyber Physical Society, Ministry of Education, Chongqing, China; 2 College of Computer Science, Chongqing University, Chongqing, China; 3 Key Laboratory of Biorheological Science and Technology, Ministry of Education, Chongqing, China; 4 College of Bioengineering, Chongqing University, Chongqing, China; National Institute of Genomic Medicine, Mexico

## Abstract

Prediction of proteasomal cleavage sites has been a focus of computational biology. Up to date, the predictive methods are mostly based on nonlinear classifiers and variables with little physicochemical meanings. In this paper, the physicochemical properties of 14 residues both upstream and downstream of a cleavage site are characterized by *VHSE* (principal component score vector of hydrophobic, steric, and electronic properties) descriptors. Then, the resulting *VHSE* descriptors are employed to construct prediction models by support vector machine (SVM). For both *in vivo* and *in vitro* datasets, the performance of *VHSE*-based method is comparatively better than that of the well-known PAProC, MAPPP, and NetChop methods. The results reveal that the hydrophobic property of 10 residues both upstream and downstream of the cleavage site is a dominant factor affecting *in vivo* and *in vitro* cleavage specificities, followed by residue’s electronic and steric properties. Furthermore, the difference in hydrophobic potential between residues flanking the cleavage site is proposed to favor substrate cleavages. Overall, the interpretable *VHSE*-based method provides a preferable way to predict proteasomal cleavage sites.

## Introduction

The ubiquitin-proteasome pathway (UPP) of protein degradation plays important roles in the cytosol and nucleus of eukaryotic cells e.g. removing misfolded, mutant, and damaged proteins [1], regulating the concentrations of regulatory proteins [2], [3], digesting foreign and native proteins into small peptides and then participating in the initiation of adaptive immune response [4].

In eukaryotic cells, the most common form of proteasome is known as the 26S proteasome, which is composed of a 20S core particle capped by a 19S regulatory particle at one or both ends [5]. The 20S core particle is a stack of four heptameric rings, which are assembled to form a cylindrical structure [6]. The outer two rings are made of α subunits (α_1_∼α_7_), which provide anchor sites for the 19S regulatory particle. The inner two rings are composed of β subunits (β_1_∼β_7_), which form proteolytic active sites in a central cavity. Three catalytic activities located in β_1_, β_2_, and β_5_ subunits are identified: peptidylglutamyl-peptide hydrolytic activity (cleavage after acidic residues); trypsin-like activity (cleavage after basic residues); and chymotrypsin-like activity (cleavage after hydrophobic residues) [7]. When cells are stimulated with pro-inflammatory cytokines, the β_1_, β_2_, and β_5_ catalytic subunits can be replaced by three new catalytic subunits: β_1i_, β_2i_, and β_5i_, respectively. This new form of proteasome is called immunoproteasome, as opposed to the constitutively expressed proteasome [8].

In the process of antigen presentation, the proteasomes can degrade proteins into peptides with 8∼12 residues [9]. It has been proved that in most circumstance, the cleavage by proteasomes only generates the C-terminus of antigens, and the N-terminals of antigens are mainly trimmed by the peptidases in cytosol or endoplasmic reticulum (ER) [10], [11]. Up to date, predictions of proteasomal cleavage sites have attracted considerable interests in computational biology. Three publicly available methods: PAProC [12], [13], MAPPP [14], [15], and NetChop [16] have been developed for predictions of proteasomal cleavage sites.

PAProC is a method for predicting cleavage sites by human proteasomes as well as wild-type and mutant yeast proteasomes. The influences of amino acids at different positions are assessed by using a stochastic hill-climbing algorithm based on the experimentally *in vitro* verified cleavage and non-cleavage sites; MAPPP is a method that combines proteasome cleavage predictions with MHC-binding predictions. FragPredict is a part of the MAPPP package that deals with the proteasome cleavage predictions. It consists of two algorithms. The first one uses a statistical analysis of cleavage -enhancing and -inhibiting amino acid motifs to predict potential proteasomal cleavage sites. The second one is based on a kinetic model of the 20S proteasome and takes the time-dependent degradation into account. This algorithm uses the results of the first algorithm as an input, and predicts which fragments are most likely to be generated. NetChop uses an artificial neural-network model that was built upon 18-residue peptide fragments consisting of full-length MHC-I ligands (9 residues) and the most proximal 9 residues flanking the C-terminus. At present, NetChop is known as the most successful method in cleavage site predictions. There are two versions of NetChop available, i.e. 1.0 and 2.0, and the later version is trained on a dataset 3 times larger than the 1.0 version. By comparing the predictive performance of PAProC, MAPPP, and NetChop, Saxova et al. [17] suggested that the predictions can still be improved, particularly if more degradation data become available.

Nussbaum et al. [18] demonstrated that certain amino acid characteristics in the positions flanking a cleavage site guide the selection of P1 residues by three active β subunits. Yael et al. [19] suggested that each position near the cleavage site contributes independently to the cleavage signal, and their contributions may be added. In light of these two points, 2607 MHC-I ligands from AntiJen database [20] and 489 *in vitro* digested data from IEDB database [21], are employed to construct a sequence-based prediction method. Characterized by *VHSE* amino acid descriptors [22], the physicochemical features of 14 residues upstream and downstream of the cleavage sites are used to establish prediction models by support vector machine (SVM). The *in vivo* and *in vitro* SVM models are further validated by two independent datasets (231 CTL epitopes and 48 *in vitro* degradation data [17]), respectively. The results show that the *VHSE*-based method is significantly superior to the well-known PAProC, FragPredict, and NetChop methods, in the consideration of predictive power and interpretability.

## Materials and Methods

### MHC-I Ligand Dataset

**Figure 1:**
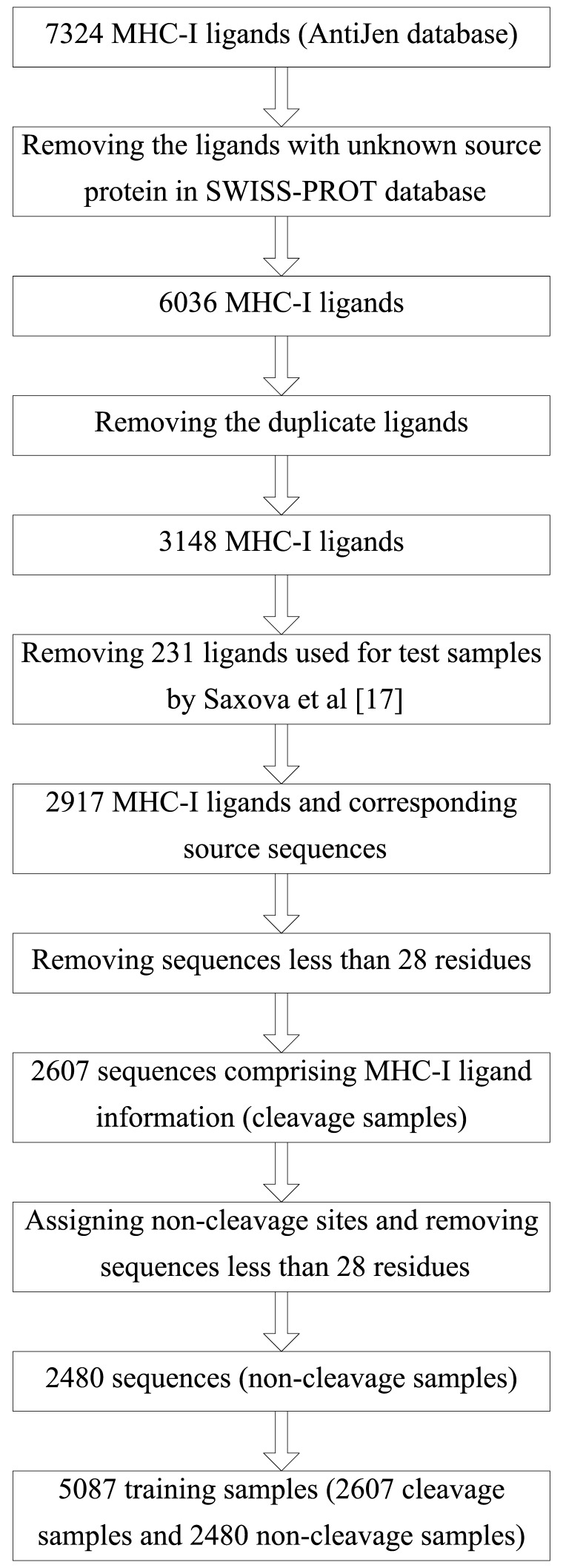
The pretreatments of the MHC-I ligands.

7324 MHC-I ligands associated with 230 human MHC-I alleles are extracted from the AntiJen database [20] (Dataset S1). The source protein sequences of these ligands are queried from the SWISS-PROT database [23]. The 7324 MHC-I ligands are pretreated according to the procedure in Figure 1 and total 2607 cleavage samples are obtained. The residues from N-terminal to C-terminal are denoted as Pn … P1 | P1' … Pn' (n = 14). The symbol “|” represents a cleavage site and the C-terminal of each MHC-I ligand is assigned as P1 position. In brief, the sequence with a span of ±14 residues from a cleavage site forms a cleavage sample.

For each cleavage sample, the middle position of the MHC-I ligand is assigned as a non-cleavage site. Thus, the sequence with a span of ±14 residues from this non-cleavage site forms a non-cleavage sample. After removing sequences less than 28 residues, total 2480 non-cleavage samples are obtained. Overall, total 5087 training samples comprising 2607 cleavage samples and 2480 non-cleavage samples are then used for SVM modeling (Dataset S2).

### *In vitro* Cleavage Dataset

857 *in vitro* cleavage products come from IEDB database [21] (Dataset S3). These peptides with 8∼11 amino acid residues are mainly from human respiratory syncytial virus (RSV) and koi herpes virus (KHV). The source protein sequence of each peptide is queried from the NCBI database [24]. The pretreatment method is the same as the MHC-I ligands. Finally, total 978 *in vitro* training data comprising 489 cleavage samples and 489 non-cleavage samples are obtained for SVM modeling (Dataset S4).

### Test Datasets

Two datasets from Saxova et al. [17] are used to validate the predictive power of the *in vivo* and *in vitro* SVM models, respectively. The first dataset comprises 231 MHC-I ligands, which are either known T cell epitopes or naturally processed peptides eluted from MHC molecules (Dataset S5 and S6). The second dataset includes 48 sequences which are digested from SSX-2 [25], HIV-Nef [26], and RUI proteins [27] by the human proteasomes (Dataset S7 and S8).

### *VHSE* Structural Description

*VHSE* (principal component score vector of hydrophobic, steric, and electronic properties), a set of amino acid descriptors comes from Mei et al. [22]. A total of 18 hydrophobic properties, 17 steric properties, and 15 electronic properties of 20 natural amino acids are used for constructing *VHSE* descriptors by principal components analysis (PCA) [22], respectively. All physicochemical properties are auto-scaled prior to PCA analysis (SPSS 10.0). For the matrices of hydrophobic, steric, and electronic properties, the first 2, 2, and 4 principal components account for 74.33, 78.68, and 77.9% variances of original property matrices, respectively. These eight principal components can be used for characterizing 20 amino acids with less information loss. The eight score vectors are so-called *VHSE* descriptors, in which *VHSE_1_* and *VHSE_2_* are related to hydrophobic properties, *VHSE_3_* and *VHSE_4_* to steric properties, and *VHSE_5_*∼*VHSE_8_* to electronic properties (Table 1).

**Table 1:** *VHSE* descriptors for 20 natural amino acids.

AA	*VHSE_1_*	*VHSE_2_*	*VHSE_3_*	*VHSE_4_*	*VHSE_5_*	*VHSE_6_*	*VHSE_7_*	*VHSE_8_*
Ala A	0.15	−1.11	−1.35	−0.92	0.02	−0.91	0.36	−0.48
Arg R	−1.47	1.45	1.24	1.27	1.55	1.47	1.30	0.83
Asn N	−0.99	0.00	−0.37	0.69	−0.55	0.85	0.73	−0.80
Asp D	−1.15	0.67	−0.41	−0.01	−2.68	1.31	0.03	0.56
Cys C	0.18	−1.67	−0.46	−0.21	0.00	1.20	−1.61	−0.19
Gln Q	−0.96	0.12	0.18	0.16	0.09	0.42	−0.20	−0.41
Glu E	−1.18	0.40	0.10	0.36	−2.16	−0.17	0.91	0.02
Gly G	−0.20	−1.53	−2.63	2.28	−0.53	−1.18	2.01	−1.34
His H	−0.43	−0.25	0.37	0.19	0.51	1.28	0.93	0.65
Ile I	1.27	−0.14	0.30	−1.80	0.30	−1.61	−0.16	−0.13
Leu L	1.36	0.07	0.26	−0.80	0.22	−1.37	0.08	−0.62
Lys K	−1.17	0.70	0.70	0.80	1.64	0.67	1.63	0.13
Met M	1.01	−0.53	0.43	0.00	0.23	0.10	−0.86	−0.68
Phe F	1.52	0.61	0.96	−0.16	0.25	0.28	−1.33	−0.20
Pro P	0.22	−0.17	−0.50	0.05	−0.01	−1.34	−0.19	3.56
Ser S	−0.67	−0.86	−1.07	−0.41	−0.32	0.27	−0.64	0.11
Thr T	−0.34	−0.51	−0.55	−1.06	0.01	−0.01	−0.79	0.39
Trp W	1.50	2.06	1.79	0.75	0.75	−0.13	−1.06	−0.85
Tyr Y	0.61	1.60	1.17	0.73	0.53	0.25	−0.96	−0.52
Val V	0.76	−0.92	0.17	−1.91	0.22	−1.40	−0.24	−0.03

In order to reduce the number of variables, only *VHSE_1_*, *VHSE_3_*, and *VHSE_5_*, i.e. the first principal component score of each matrix are used for structural characterizations of cleavage/non-cleavage samples. For example, a sample with 14 residues on either side of the cleavage site (±14) can now be characterized by 28×3 = 84 *VHSE* variables.

### Support Vector Machine (SVM)

As a supervised learning method for classification, SVM [28], [29] was originally proposed for solving the classification problem of linearly divisible samples. The core idea of SVM is to find an optimal separating hyperplane, which maximizes the distance of either class to this hyperplane, and minimizes the risk of misclassification. For nonlinear classification problem, SVM performs a nonlinear mapping from an input space to a high-dimensional feature space, and then applies linear classification techniques in this high-dimensional space. The nonlinear mapping is accomplished by a kernel function: *K(x,x_i_) = Φ(x)·Φ(x_i_)*. By introducing kernel functions, SVM can effectively avoid the problems of over-fitting, dimension disaster, and local optimum. Below are some useful kernel functions:
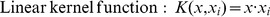
(1)

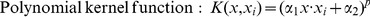
(2)

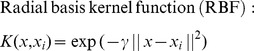
(3)

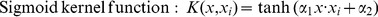
(4)


According to our experience and previous researches [30]–[32], the RBF kernel is usually superior to other non-linear kernel functions. Therefore, only linear and RBF kernels are used for SVM modeling. In this paper, SVM is implemented by SVM_light program [33]. Each *VHSE* variable is scaled linearly to [0, 1] before SVM modeling. The optimal values of C, ε and γ are determined by the results of 10-fold cross-validation.

### Measures of Performance

The performance of SVM models is evaluated by accuracy (*Acc*), sensitivity (*Sen*), specificity (*Spe*), and Matthew’s correlation coefficient (*MCC*), the definitions of which are shown in Equation 5∼8.
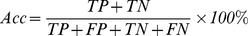
(5)

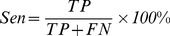
(6)

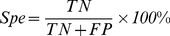
(7)

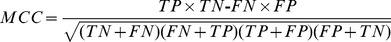
(8)Where *TP* is the number of true positives; *TN* is the number of false positives; *FP* is the number of true negatives and *FN* is the number of false negatives. The *MCC* is a balanced measure which can be used even if the classes are of very different sizes [34]. The area under receiver operating characteristics curve (*AUC*), a global threshold-independent measure of performance, is also used for model evaluations [35].

## Results and Discussion

### SVM Modeling

In order to examine the influence of sequence length on model performance, training samples with a span of ±6, ±8, ±10, ±12, and ±14 residues from cleavage/non-cleavage sites are used to construct SVM models, respectively. The performance of the SVM models are shown in Table 2. For both *in vivo* and *in vitro* datasets, the model performance increases with the sequence length in the range of ±6∼±10. However, the performance begins to decrease when the sequence length is beyond ±10 residues. The results imply that residues outside the range of ±10 have little contributions to substrate cleavages. Meanwhile, no significant difference is observed between linear and RBF kernels. In the consideration of complexity and interpretability, the linear SVM models are selected as the optimal models for both datasets, denoted by SVM_MHC-I_ and SVM_VITRO_, respectively.

**Table 2:** Performance of SVM models.

Dataset 1: MHC-I ligands						
Sequence length	Kernel	*MCC*	*AUC*	*Acc (%)*	*Sen (%)*	*Spe (%)*
±6 (12)	Linear	0.5419	0.8457	77.11	78.86	75.28
	RBF	0.5411	0.8459	77.07	78.85	75.20
±8 (16)	Linear	0.5677	0.8586	78.35	82.66	73.83
	RBF	0.5676	0.8591	78.35	82.54	73.95
±10 (20)	Linear^a^	0.5905	0.8673	79.52	82.74	76.13
	RBF	0.5902	0.8691	79.52	82.20	76.69
±12 (24)	Linear	0.5842	0.8701	79.22	81.78	76.53
	RBF	0.6082	0.8809	80.42	82.93	77.78
±14 (28)	Linear	0.5803	0.8705	79.02	81.85	76.04
	RBF	0.5896	0.8746	79.49	81.74	77.13
**Dataset 2: ** ***in vitro*** ** cleavage data**						
**Sequence length**	**Kernel**	***MCC***	***AUC***	***Acc (%)***	***Sen (%)***	***Spe (%)***
±6 (12)	Linear	0.5099	0.8345	75.45	78.12	72.78
	RBF	0.5162	0.8357	75.76	78.74	72.78
±8 (16)	Linear	0.5265	0.8380	76.27	79.34	73.20
	RBF	0.5092	0.8364	75.44	75.86	75.03
±10 (20)	Linear^b^	0.5481	0.8310	77.39	76.68	78.09
	RBF	0.5399	0.8318	76.98	76.88	77.08
±12 (24)	Linear	0.5174	0.8377	75.85	76.26	75.45
	RBF	0.5338	0.8368	76.67	75.65	77.69
±14 (28)	Linear	0.5318	0.8354	76.57	75.86	77.29
	RBF	0.5358	0.8392	76.79	77.10	76.48

The predictive power of SVM_MHC-I_ and SVM_VITRO_ are further validated by two independent test sets provided by Saxova et al. [17], respectively. The overall predictive accuracies for SVM_MHC-I_ and SVM_VITRO_ model are 73.5% and 70.5%, respectively (Table 3). It is clear to see that the predictive power of SVM_MHC-I_ and SVM_VITRO_ are significantly better than that of PAProC, MAPPP, NetChop 1.0 and 2.0, especially in the level of *MCC*. Why our models generate more reliable predictions? There are 3 main reasons. Firstly, more training samples are involved in the SVM modeling. NetChop 2.0 is trained on 1110 MHC-I ligands, whereas SVM_MHC-I_ on 2607 MHC-I ligands. Secondly, more residues, i.e. a span of ±10 residues from the cleavage site, are considered in our models. Lastly, SVM_MHC-I_ and SVM_VITRO_ are established by SVM technique, which has better generalization capability and extendibility than the artificial neural network adopted by NetChop. However, the most important thing is that SVM_MHC-I_ and SVM_VITRO_ outperform the other models in model’s interpretability. Following is a detailed analysis of proteasomal cleavage specificities based on SVM_MHC-I_ and SVM_VITRO_ models.

**Table 3:** The predictive power of SVM_MHC-I_ and SVM_VITRO_ in comparison with the other 4 models.

	Test set 1: MHC-I ligands				Test set 2: *In vitro* data			
Model	*Acc(%)*	*Sen (%)*	*Spe (%)*	*MCC*	*Acc(%)*	*Sen(%)*	*Spe(%)*	*MCC*
aPAProC	NA^b^	45.6	30.0	−0.25	NA	46.4	64.7	0.10
aFragPredict	NA	83.5	16.5	0.00	NA	72.1	41.4	0.12
aNetChop1.0	NA	39.8	46.3	−0.14	NA	34.4	91.4	0.31
aNetChop2.0	NA	73.6	42.4	0.16	NA	57.4	76.4	0.32
SVM_MHC-I_	73.5	82.3	64.8	0.48				
SVM_VITRO_					70.5	62.5	78.7	0.42

a The predictive performance of PAProC, FragPredict, NetChop1.0 and 2.0 are cited from Saxova et al. [17].

b Not available.

### *In vivo* Cleavage Specificities of Proteasome

**Figure 2:**
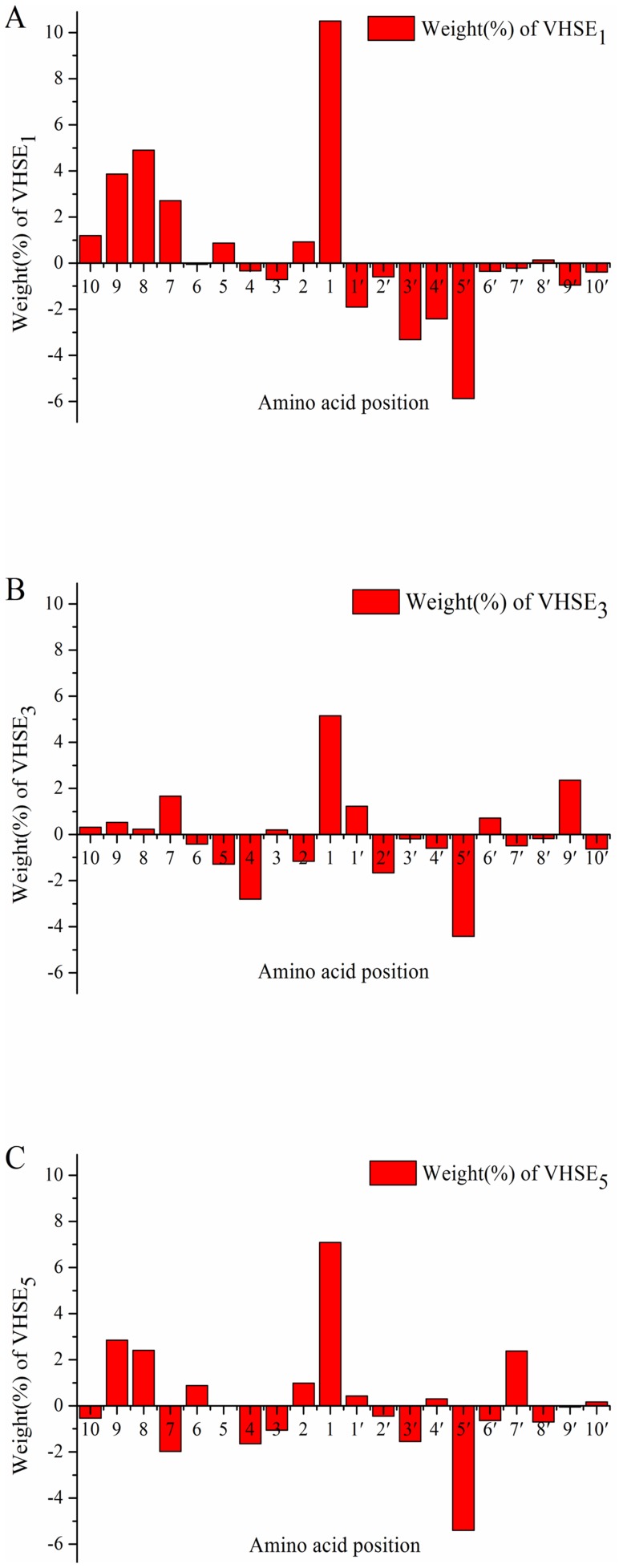
The weight coefficients of *VHSE* variables included in SVM_MHC-I_ model. A: *VHSE*_1_ (Hydrophobic property); B: *VHSE*_3_ (Steric property); C: *VHSE*_5_ (Electronic property).

From the sequence information of proteasomal degradation products, it has become clear that the nature of the proteasome target sites cannot explain the cleavage specificities alone and the sequence context adjacent to a cleavage sites also play an important role [36]–[38]. From the results of SVM modeling, it can be indicated that ±10 residues upstream and downstream of a cleavage site contribute to both the *in vivo* and *in vitro* cleavage specificities. The SVM_MHC-I_ model is trained on naturally processed MHC-I ligands, thus, it can reflect the *in vivo* cleavage specificities of proteasomes. Figure 2 is the plot of weight coefficients of *VHSE* variables involved in SVM_MHC-I_. For convenience, the weight coefficients of *VHSE_1_*, *VHSE_3_*, and *VHSE_5_*, which characterize hydrophobic, electronic, and steric properties, are shown in Figure 2A, 2B, and 2C, respectively. Overall, the hydrophobic, electronic, and steric properties of residues are closely related to the cleavage specificities, especially for P9, P8, P7, P4, P1, P3', P4', and P5' positions.

As shown in Figure 2A, *VHSE_1_* variable at the P1 position has the largest positive weight coefficient (10.49). That is to say, the P1 position prefers hydrophobic residues. Falk et al. [39] found that hydrophobic Leu, Ile, Val, Thr, and Ala are the most abundant residues at the C-terminal (P1) of antigenic peptides. Earlier researches also indicated that the degradation products with hydrophobic C-terminal residues can be easily transferred to ER and bind to MHC molecules [40], [41]. These are consistent with our results.

Besides P1 position, the weight coefficients of *VHSE_1_* upstream of the cleavage site are mainly positive, such as P2, P5, P7, P8, P9 and P10. However, the weight coefficients of *VHSE_1_* variables downstream of the cleavage site, except for P8', are negative. Namely, there is a significant difference in the weight coefficients of *VHSE_1_* between positions upstream and downstream of the cleavage sites. So, it can be inferred that hydrophobic potential flanking the cleavage site is beneficial for substrate hydrolysis.

*In vitro* experiments showed that Leu|Lys is a strong cleavage site [38]. According to *VHSE_1_* values of Leu (1.36) and Lys (−1.17) together with the weight coefficient for each position, it can be inferred that Leu|Lys is a favorable combination for proteasomal cleavage.

From Figure 2B, it can be seen that that the *VHSE_3_* variables (steric property) of P1, P5', P4 and P9' positions have more influence on cleavage specificities. For P5' and P4 positions with negative *VHSE_3_* weight coefficients, bulky residues are unfavorable to substrate cleavages. Nussbaum et al. [18] also proved that a small Pro is the most preferred at the P4 position for wild-type yeast 20S proteasome.

According to the weight coefficients of *VHSE_5_* (Figure 2C), electronic properties of residues at P1, P5', P9, P8, and P7' exert more influence on the cleavage specificities. Nussbaum et al. [18] observed that polar residues at P5' and P3 positions are clearly favored over non-polar ones for β5 active site, which is agreement with our results.

In general, the *VHSE* weight coefficients of P1, P8, and P9 positions are very similar to each other. These three positions are all inclined to select hydrophobic, bulky, and electro-positive residues. Also, the *VHSE* weight coefficients are similar for P2', P3', and P5', which tend to select hydrophilic, small, and electro-negative residues. Interestingly, the preferences of P2', P3', and P5' are directly opposite to that of P1, P8, and P9. The profiles of *in vivo* cleavages are summarized in Table 4.

**Table 4:** The profiles of *in vivo* cleavages.

Position	Favored^a^	Unfavored^b^
P9	F, W, L, M	E, D, N, S
P8	F, W, L, I	R, E, K, D
P7	F, W, L, I	R, E, K, D
P4	G, A, S	W, R, Y
P1	F, W, K, R, I	E, D, N, T
P3'	R, E, K, D	F, W, L, I
P4'	R, E, K, D	F, W, L, I
P5'	E, D, N, T	F, W, K, R, I

a The residues in the corresponding positions are favorable to substrate cleavages;

b The residues in the corresponding positions are unfavorable to substrate cleavages.

### *In vitro* Cleavage Specificities of Proteasome

**Figure 3:**
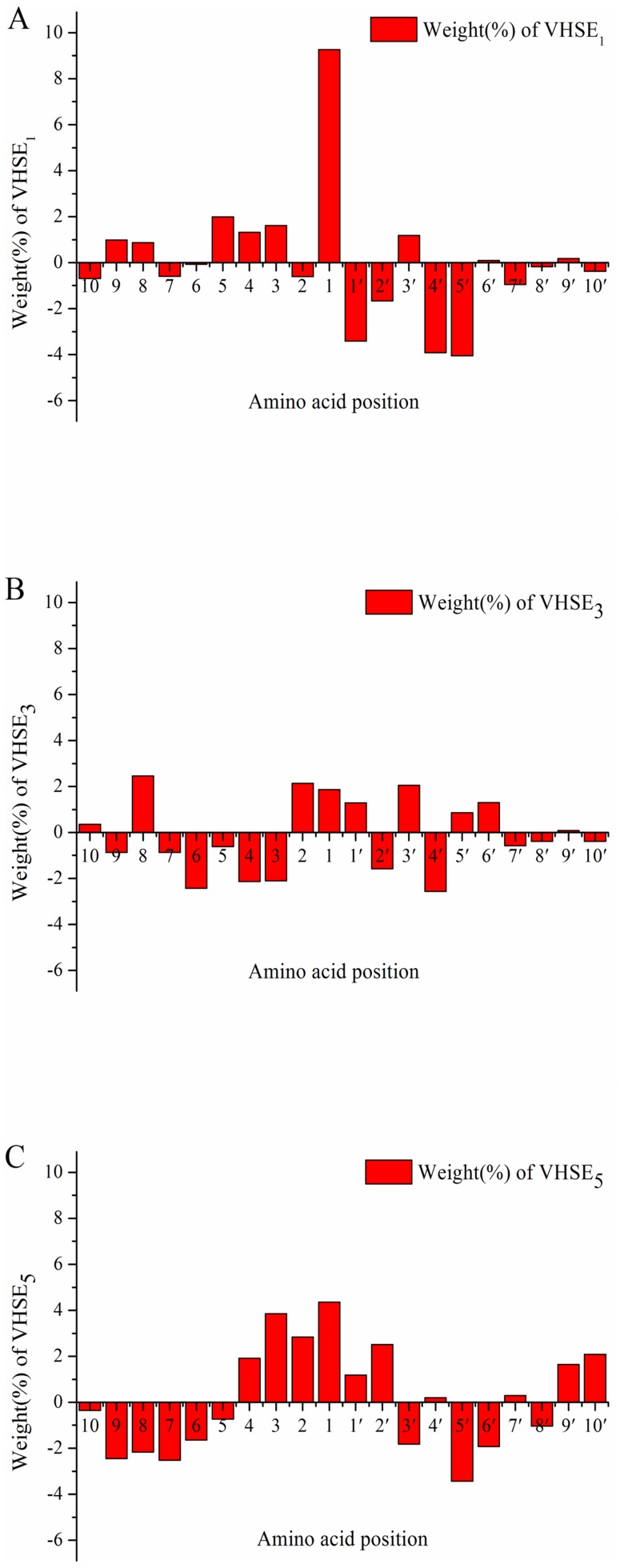
The weight coefficients of *VHSE* variables included in SVM_VITRO_ model. A: *VHSE*_1_ (Hydrophobic property); B: *VHSE*_3_ (Steric property); C: *VHSE*_5_ (Electronic property).

Compared with SVM_MHC-I_, the SVM_VITRO_ model based on experimental *in vitro* data reflects *in vitro* cleavage specificities of proteasomes. Due to the differences between *in vivo* cellular environment and *in vitro* cell-free system, the cleavage specificities of proteasomes should be somewhat different. For reasons of convenience, the weight coefficients of *VHSE*_1_, *VHSE*_3_, and *VHSE*_5_ for the SVM_VITRO_ model are shown in Figure 3A, 3B, and 3C, respectively.

As was the case with the *in vivo* SVM_MHC-I_ model, P1 position exerts the most important influence on the proteasomal cleavage, as shown in Figure 3. It is clear to see that *VHSE*_1_ (hydrophobic) at the P1 position is a dominant variable affecting proteasomal cleavage. For P7, P8, and P9 positions, the *VHSE*_1_ variables have relatively less influence on the proteasomal cleavage in comparison with the case of SVM_MHC-I_. Except for P3', the weight coefficients of the *VHSE*_1_ variables downstream of the cleavage site are similar to the case of SVM_MHC-I_. Taken as a whole, hydrophobic potential difference flanking the cleavage sites is also beneficial to the *in vitro* proteasomal cleavages.

The contribution of *VHSE*_3_ (steric) to the proteasomal cleavages is less than that of *VHSE*_1_ (Figure 3B). Compared with the case of SVM_MHC-I_ (Figure 2B), no significant steric hindrance effect is observed for residues in the vicinity of the cleavage site, which may be caused by the absence of cell environment.

Significant difference in the weight coefficients of *VHSE*_5_ (electronic) is observed between the case of SVM_VITRO_ (Figure 3C) and SVM_MHC-I_ (Figure 2C). Interestingly, the signs of *VHSE*_5_ weight coefficients in SVM_VITRO_ seem to vary in an interval of 6 residual positions (Figure 3C)_._ Compared with the case of SVM_MHC-I_, the influence of *VHSE*_5_ at P1 and P5' positions on the substrate cleavages decreases significantly, while the influence of P2, P3, and P2' increases.

Overall, hydrophobic and electronic properties have more impact than steric properties on selection specificities in the *in vitro* system.

### Conclusion

Based on SVM classification technology and *VHSE* description method, QSAR models with excellent predictive power are established for predicting proteasomal cleavage sites. The results show that hydrophobic property of residues flanking the cleavage site is a dominant factor affecting both the *in vivo* and *in vitro* cleavage specificities, followed by electronic and steric properties. The difference in hydrophobic potential between residues upstream and downstream of the cleavage sites is proposed to favor the substrate cleavages, especially for *in vivo* cleavages. For the *in vivo* SVM_MHC-I_ model, the hydrophobic properties of the P1, P8, P9, and P5' play more important roles than that of other positions. In addition, the electronic and steric properties of P1 and P5' positions also have a great impact on the substrate cleavages. In comparison with the case of SVM_MHC-I_, the influence of residue’s hydrophobic and steric properties on substrate cleavages seems to decrease in the case of SVM_VITRO_. However, the contribution of residue’s electronic properties increases significantly, probably due to the solvation effect of the cell-free system.

In summary, compared to the well-known PAProC, FragPredict, and NetChop methods, the SVM_MHC-I_ and SVM_VITRO_ models are trained on larger datasets and have preferable predictive performance and interpretability. The studies presented in this paper would facilitate a deep understanding of the *in vivo* and *in vitro* selective cleavages as well as the cleavage mechanisms of the proteasomes.

## Supporting Information

Dataset S1 **The original data of MHC-I ligands.** This excel workbook presents the 7324 MHC-I ligands extracted from the AntiJen database.
(XLSX)

Dataset S2 **The resulting** ***VHSE*** **descriptors of 5087** ***in vivo*** **samples used for SVM modeling.**
(XLS)

Dataset S3 **The original data of** ***in vitro*** **proteasomal cleavage.** This excel workbook presents the 857 *in vitro* cleavages products derived from the IEDB database.
(XLS)

Dataset S4 **The resulting** ***VHSE*** **descriptors of 978** ***in vitro*** **samples used for SVM modeling.**
(XLS)

Dataset S5 **The first test set.** This dataset contains 231 MHC-I ligands, which are either know T cell eptiopes or naturally processed peptides eluted from MHC molecules.
(XLSX)

Dataset S6 **The resulting** ***VHSE*** **descriptors of the first test samples.**
(XLS)

Dataset S7 **The second test set.** This dataset contains 48 products of peptide degradation by the human constitutive proteasome *in vitro*.
(XLSX)

Dataset S8 **The resulting VHSE descriptors of the second test samples.**
(XLSX)
